# Predicting clinical resistance prevalence using sewage metagenomic data

**DOI:** 10.1038/s42003-020-01439-6

**Published:** 2020-11-26

**Authors:** Antti Karkman, Fanny Berglund, Carl-Fredrik Flach, Erik Kristiansson, D. G. Joakim Larsson

**Affiliations:** 1 Department of Microbiology, University of Helsinki, Helsinki, Finland; 2 Helsinki Institute of Sustainability Science, University of Helsinki, Helsinki, Finland; 3 Department of Infectious Diseases, Institute of Biomedicine, Sahlgrenska Academy, University of Gothenburg, Gothenburg, Sweden; 4 Centre for Antibiotic Resistance Research (CARe), University of Gothenburg, Gothenburg, Sweden; 5 Department of Mathematical Sciences, Chalmers University of Technology and University of Gothenburg, Gothenburg, Sweden

**Keywords:** Bacterial infection, Water microbiology, Antimicrobial resistance

## Abstract

Antibiotic resistance surveillance through regional and up-to-date testing of clinical isolates is a foundation for implementing effective empirical treatment. Surveillance data also provides an overview of geographical and temporal changes that are invaluable for guiding interventions. Still, due to limited infrastructure and resources, clinical surveillance data is lacking in many parts of the world. Given that sewage is largely made up of human fecal bacteria from many people, sewage epidemiology could provide a cost-efficient strategy to partly fill the current gap in clinical surveillance of antibiotic resistance. Here we explored the potential of sewage metagenomic data to assess clinical antibiotic resistance prevalence using environmental and clinical surveillance data from across the world. The sewage resistome correlated to clinical surveillance data of invasive *Escherichia coli* isolates, but none of several tested approaches provided a sufficient resolution for clear discrimination between resistance towards different classes of antibiotics. However, in combination with socioeconomic data, the overall clinical resistance situation could be predicted with good precision. We conclude that analyses of bacterial genes in sewage could contribute to informing management of antibiotic resistance.

## Abstract (web summary)

Karkman et al. explore how well available global sewage metagenomic data can predict clinical resistance prevalence using different models. A combination of sewage metagenomic data with socioeconomic factors predicts overall clinical resistance well, but still has limited ability to discriminate between resistance to different classes of antibiotics.

## Introduction

Antibiotics are critical for the prevention and treatment of bacterial infections. The global rise in antibiotic resistance is therefore threatening large parts of modern health care. Regional, up-to-date surveillance data on resistance levels to different antibiotics in different pathogens is critical for guiding optimal empirical antibiotic treatment^1^. Surveillance also serves the purpose of identifying temporal resistance trends that can be used to guide or evaluate interventions to reduce the development of multiresistant bacteria.

Traditional surveillance data are based on data from a large number of clinical isolates, each subjected to susceptibility testing with standardized methods. As this approach is labor intensive, expensive and needs specialized infrastructure^2^, surveillance data are lacking or very limited in many parts of the world, not least in low- and middle-income countries^3^. In addition, the collection of resistance data from clinical isolates is far from standardized across the world^4^, making comparisons between countries challenging and hampering global efforts to tackle the resistance crisis.

For decades, municipal sewage has been used to monitor poliovirus at a community level^5^. More recently, a similar approach has been taken to capture regional development of SARS-CoV-2 prevalence^6,7^. Sewage epidemiology has also been proposed as an alternative or complementary method to overcome some of the challenges in antibiotic resistance surveillance, particularly the need for extensive patient sampling and the limited resources and infrastructure in low- and middle-income countries. In addition, sewage epidemiology can be used to sample entire populations without linking data to individuals, avoiding ethical challenges related to traditional surveillance^1,2,4^ and even serve as an early warning system for new or rare forms of antibiotic resistance^8,9^. Clinical resistance surveillance is often biased to more severe cases as only a minority of non-hospitalized patients are being sampled and samples taken after initial treatment failure are overrepresented^2^. In contrast, sewage-based surveillance covers the whole population served by the sewage network and therefore presents a less-biased picture of regional resistance levels^8–11^. The differences in resistance prevalence in hospital and community waste waters also demonstrate the bias in clinical settings^2,8,9^.

There are at least two main approaches for sewage-based antibiotic resistance monitoring; isolate-based approaches or gene-based analyses of complex microbial communities. Some of the advantages and disadvantages of both approaches have recently been reviewed^12^. In short, the isolate-based approach uses susceptibility testing of many individual bacterial isolates and thus offers direct information about the resistance phenotype, including multi-resistance patterns in clinically relevant species. However, although a large number of isolates can be efficiently collected and managed as recently indicated by a global monitoring of cefotaxime-resistant coliforms from sewage^13^, this approach can be as labor intensive as clinical surveillance with regard to the susceptibility testing part. The gene-based approach, on the other hand, uses the metagenomic DNA of the sewage community and can either target the whole resistome (shotgun sequencing) or focus on a specific set of resistance genes (PCR). Therefore, the gene-based approach reflects the genetic basis for resistance in all species present in the sewage, but cannot offer direct information on resistance phenotype. The infrastructure for sequencing and bioinformatic analyses could easily be centralized and only sampling would need to be done locally, further reducing the costs and infrastructure needed and making the method easier to standardize^4^. There are, however, several conceptual limitations with a metagenomic approach. These include (1) prediction from genotype to phenotype (2) challenge to identify point mutations leading to antibiotic resistance, (3) shotgun sequencing makes it hard to assign resistance genes and thus predicted resistances to specific species, and (4) the challenge to identify multi-resistance patterns without data on isolates.

Depending on the intended purpose of surveillance and thus the need for precision, metagenomic analyses may still prove to be valuable, but until now, an evaluation of how well such data correlates to regional clinical surveillance data has been lacking^4^. Some data do suggest that it could be possible to predict clinical resistance levels from sewage data. In a trans-European high-throughput PCR-array based surveillance study, the abundance of sewage antibiotic resistance genes (ARGs) was shown to mirror the north-to-south clinical resistance gradient in Europe^14^. Furthermore, resistance levels of *Escherichia coli* isolated from sewage have recently been shown to correlate with the resistance levels in clinical isolates^1,2^.

Recently, a global monitoring of ARGs in sewage was conducted, including over 60 countries^15^. There were systematic differences in ARG prevalence between continents; Asia, Africa, and South America having a higher incidence of ARGs in sewage compared with Europe, North-America, and Oceania. Antibiotic usage is often considered to be the most important factor driving resistance, but in this study the differences in sewage resistance could be best explained with socioeconomic factors^15^. Another study came to similar conclusions when analyzing global clinical resistance data; antibiotic use was not the best predictor, but rather socioeconomic factors^16^. Both Collignon et al.^16^ and Hendriksen et al.^15^ observed that factors related to sanitation/hygiene are significantly associated with antibiotic resistance, both when expressed in terms of ARG abundances in sewage and when expressed in terms of the proportion of resistant *E. coli* in clinical isolates. Thus, both studies hypothesized that contagion, the spread of resistant bacteria, could be one of the most important drivers of the prevalence of clinical resistance globally. Poor hygiene, poor sanitation, and poor infrastructure enhance contagion, resulting in more individuals being infected with/carriers of resistant strains^17^. This could, in turn, lead to increased shedding of resistant bacteria on a community level, which could be reflected in sewage. Resistance in sewage bacteria is, conceptually, a more direct measure of resistance in a population than both antibiotic use or social factors, mirroring insufficient transmission control. Data from sewage therefore has the potential to reflect clinical resistance levels regardless of drivers, and possibly better than previous models based on social factors.

As metagenome-based surveillance data quantify the whole-community resistome, it is not obvious which genetic markers correlate best with clinical resistance to the different classes of antibiotics in infections caused by a given pathogen. An optimal marker should be both specific and sensitive to be able to accurately reveal the local clinical resistance levels in a given species to a given antibiotic. One apparent approach would be to quantify ARGs for each class of antibiotic in sewage metagenomes separately, but given that ARGs are unequally distributed across species, this might be misleading when correlating the sum of ARGs to the resistance in clinical isolates of a given species. A way to at least partly circumvent this could be to use only ARGs commonly found in a given species, e.g., ARGs in the *E. coli* accessory genome. Still, a large portion of the ARGs known to occur in *E. coli* may actually be present in other species in the sewage community, thus likely reducing the correlation. As a broad indication of resistance, the prevalence of class 1 integrons, more specifically the *intI1* integrase gene, has previously been linked to phenotypic resistance in *E. coli*^18,19^, hence the correlation to this gene could be worthwhile investigating.

Here, we aimed to explore whether sewage metagenomic data reflect clinical antibiotic resistance on a global scale and if so, how accurately it is possible to predict clinical resistance using models based on sewage metagenomic data. To achieve this, we investigated the correlation between reported clinical resistance prevalence in bloodstream infections of *E. coli* or an aggregated resistance index from^16^ and either (1) total ARGs, (2) total ARGs for each antibiotic class separately, (3) the 10 most abundant ARGs that are known to be present in the *E. coli* pangenome, (4) the 10 most abundant *E. coli* ARGs separated by antibiotic class, (5) *IntI1* integron gene abundances in sewage. Furthermore, we (6) compared whether sewage data correlated better to the prevalence of clinical resistance compared with socioeconomic factors. Last, we (7) explored if models based on both sewage data and socioeconomic factors would give the best model for clinical resistance prevalence in terms of model fit. After a leave-one-out cross-validation of the models, we (8) used sewage data together with socioeconomic factors to predict clinical antibiotic resistance in countries where surveillance data are lacking.

## Results and discussion

In this study, we explored the possibility to use country level relative abundance of ARGs in sewage metagenomic data to predict country level proportion of antibiotic-resistant clinical isolates. We annotated the total acquired resistance gene abundance and the class 1 integron (*intI1)* integrase gene abundance from recently published global sewage metagenomic data set from ref. ^15^. Publicly available country level data on the proportion of resistant invasive *E. coli* clinical isolates was collected based on availability for four different antibiotic classes; aminopenicillins, fluoroquinolones, third-generation cephalosporins and aminoglycosides. In addition, we used the “aggregate resistance” index as generated by Collignon et al.^16^, representing the combined average prevalence of *E. coli* and *Klebsiella spp* resistant to third-generation cephalosporins, fluoroquinolones, and carbapenems, and meticillin-resistant *Staphylococcus aureus*.

**Fig. 1:**
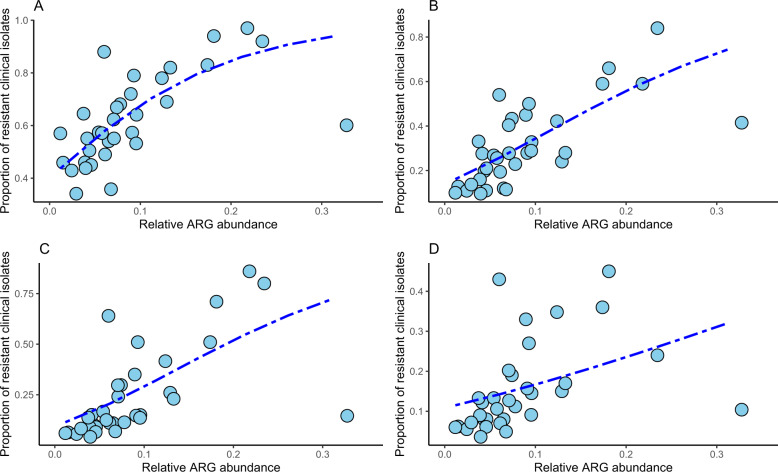
*E. coli* clinical resistance models based on the ten most common ARGs in *E. coli*. Proportion of resistant invasive *E. coli* clinical isolates to aminopenicillins **a**, fluoroquinolones **b**, third generation cephalosporins **c**, and aminoglycosides **d** against the relative abundance of the 10 most common ARGs in* E. coli.* The blue line shows the fitted clinical resistance from the beta regression model with resistance gene abundance as explanatory variable. Note that for some countries, data on clinical resistance was not available for all classes.

First, the annotated acquired ARGs in the sewage metagenomic data were correlated with the proportion of resistant clinical *E. coli* using a beta regression model. The relative abundance of all ARGs could, however, not to a large extent, explain the variability in the clinical resistance patterns (*p* value < 0.05, *R*^2^ 0.12–0.24, Table 1). A similar result was obtained when separating the ARGs into individual resistance classes (i.e., beta-lactam, fluoroquinolone, third-generation cephalosporin, and aminoglycoside resistance genes). Here, only the models where beta-lactam ARGs were correlated with clinical aminopenicillin resistance had a noticeable higher model fit than when using all combined ARGs (*p* value < 0.05, *R*^2^ 0.31, Supplementary Fig. 2 and Table 1). Next, in an effort to further reduce the noise in the data, only the ten most common acquired ARGs found in all sequenced *E. coli* genomes were used. These models showed higher model fits for all resistance classes compared with the models using all ARGs found in the metagenomic data (*R*^2^ 0.41, 0.49, 0.39, and 0.20 for the clinical aminopenicillin, fluoroquinolone, third-generation cephalosporin and aminoglycoside resistance, respectively, Fig. 1, Table 1).

**Table 1:** The obtained *R*^2^ from the beta regression analysis using the metagenomic sewage data.

	AP-res.	FQ-res	3GC-res	AG-res	Aggregated resistance
All ARGs	0.24	0.24	0.21	0.12	0.32
BL-ARGs	0.31	0.22	0.21	0.12	0.16
FQ-ARGs	0.26	0.26	0.23	0.14	0.08
Third gen. Ceph. ARGs	0.34	0.20	0.24	0.10	0.17
AG-args	0.17	0.21	0.14	0.10	0.29
*E. coli* top ten ARGs	0.41	0.49	0.39	0.20	0.30
*E. coli* top ten, no outlier	0.72	0.67	0.72	0.44	0.34
*E. coli* top ten BL-ARGs	0.48	0.38	0.46	0.16	0.07
*E. coli* top ten FQ-ARGs	0.17	0.15	0.14	0.09	0.03
*E. coli* top ten third gen. ceph. ARGs	0.53	0.25	0.43	0.12	0.21
*E. coli* top ten AG-args	0.24	0.33	0.24	0.17	0.31
*IntI1*	0.59	0.66	0.62	0.31	0.24
Combined *IntI1* + socioeconomical factors	0.85	0.80	0.86	0.68	0.76

We observed that one country, Malta, created an outlier that strongly affected the models with the ten most common *E. coli* ARGs. When Malta was excluded from the analysis, the model fit increased substantially for all clinical resistance classes (*R*^2^ values of 0.72, 0.67, 0.72, and 0.44 for the aforementioned clinical resistance profiles, Supplementary Fig. 3). However, as we had no prior reason to believe that the data obtained for Malta is erroneous, we chose to keep it in the model. When using the ten most abundant ARGs in *E. coli* separated by the antibiotic class they confer resistance to, a better model fit was achieved for the beta-lactam and third generation cephalosporin ARGs predicting aminopenicillin and third generation cephalosporin resistance (*R*^2^ values of 0.48 and 0.53, respectively). It should be noted that cross-correlations (using ARGs for one antibiotic class to correlate with clinical resistance to another class) was approximately as good, or even better (e.g., beta-lactam ARG model for clinical fluoroquinolone resistance) than within-class correlations (Table 1). Although this may appear counterintuitive, this could have multiple explanations. Even with the attempt to use only ARGs found in *E. coli*, a large portion of the ARGs could very well be present in other species in the sewage. The relative abundance of the genus *E.* *coli* in the sewage metagenomes varied from 0.1 to 10%, with a mean of 1%^15^. Our inability to capture mutation-based resistance, the dominant factor behind, e.g., fluoroquinolone resistance, is another possible reason. It should be noted that clinical resistance prevalence to the investigated antibiotic classes were strongly correlated to each other (Supplementary Fig. 1). With such close correlation, it becomes more difficult to find models that can explain resistance to individual classes of antibiotics with high specificity.

**Fig. 2:**
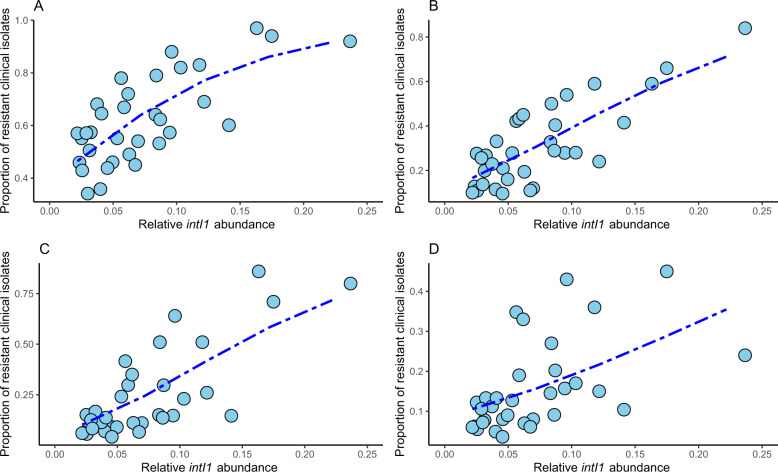
*E. coli* clinical resistance models based on the *intI1* integrase gene. Proportion of resistant invasive *E. coli* clinical isolates to aminopenicillins **a**, fluoroquinolones **b**, third-generation cephalosporins **c**, and aminoglycosides **d** against *intI1* integrase gene abundance. The blue line shows the fitted clinical resistance from the beta regression model with *intI1* abundance as explanatory variable. Note that for some countries, data on clinical resistance was not available for all classes.

Given that the class-related information on ARGs had a limited contribution to the correlations, the correlation between *intI1* integrase gene abundance and prevalence of resistance to different classes of antibiotics in clinical *E. coli* isolates was investigated. Here, the level of correlation varied substantially between antibiotic classes (*p* value < 0.01, *R*^2^ 0.31–0.66, Fig. 2, Table 1). The best model was found for fluoroquinolone (*R*^2^ 0.66) and third-generation cephalosporin (*R*^2^ 0.62) resistance in clinical isolates. The model for aminoglycoside resistance had the lowest model fit (*R*^2^ 0.31). Although total acquired ARG and *intI1* gene counts were correlated in the sewage data (*p* < 0.01, *R*^2^ 0.42, Suppl. Material), *intI1* overall performed better. The association of *intI1* to phenotypic resistance in *E. coli*^18,19^ might have less noise compared with the resistance gene approach, which is hampered by the broad distribution of ARGs in bacteria, explaining the overall better performance. This, in turn, would mean that this approach might not be optimal for other clinically relevant pathogens.

**Fig. 3:**
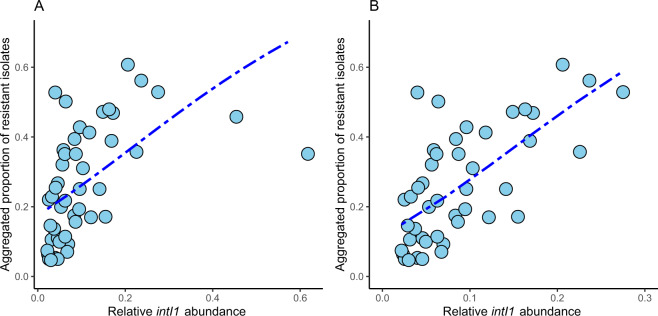
Aggregated resistance model based on *intI1* integrase gene. Aggregated resistance index as described in Collignon et al.^16^ correlated with normalized *intI1* gene abundance in sewage. All data in **a** and two outliers (Nigeria and Peru) removed in **b**. The blue line shows the fitted clinical resistance from the beta regression model with *intI1* abundance in sewage as an explanatory variable.

When modeling the aggregated resistance index, neither the intI1 abundance nor the acquired *E. coli* ARGs had as good model fit as for *E. coli* clinical resistance patterns. However, they were still significantly associated (*p* value < 0.01, *R*^2^ 0.24 and 0.30, respectively, Table 1, Fig. 3a and Supplementary Fig. 4). In the *intI1* model, two outlier data points (Nigeria and Peru) had very high *intI1* abundance, whereas the aggregated resistance index was moderate. Removing these two data points enhanced the model considerably (*p* value < 0.01, *R*^2^ 0.43, Fig. 3b). There was no clinical resistance surveillance data for *E. coli* available from these two countries. The outliers could, potentially, be caused by an error in the aggregated resistance index data since higher values, at least for Peru, were already reported in 2008–2009^20^. There is only sparse data on clinical resistance prevalence for Nigeria, but very high resistance levels for *E. coli* and *Klebsiella* against third-generation cephalosporins (80–90%) have been reported^21^.

Taken together, the total abundance of all acquired ARGs, the top ten most common *E. coli* associated acquired ARGs and *intI1* gene counts could be used to predict clinical resistance prevalence with varying precision. Sewage metagenomic data also appears to provide more accurate predictions of clinical resistance levels to some antibiotic classes than others. As *intI1* integrase gene counts overall performed the best in our analysis, and is a considerably simpler measure than a large set of ARGs, it was used in further analyses. These results should therefore be interpreted as indications of the overall resistance prevalence. Therefore, resistance patterns to any antibiotic classes that do not follow the overall resistance pattern would be predicted less accurately.

Although sewage metagenome data alone correlated with clinical resistance with reasonably high precision in most cases, there was still a lot of variability in the data that could not be explained with the sewage data alone. Socioeconomic factors have been shown to be good predictors of clinical resistance^16,17^. Previous results suggest that contagion may be one of the most important factors contributing to the prevalence of clinical resistance^17^. Countries with better sanitation and better health care infrastructure, and therefore less contagion in, e.g., hospitals, could have lower clinical resistant prevalence despite the higher resistance levels in the overall population. Since socioeconomic factors, most likely, have an indirect influence on the clinical resistance levels, the sewage data could be a more direct measure reflecting the prevalence of resistance in local populations.

To compare correlations between sewage data and prevalence of clinical resistance to correlations between socioeconomical factors and clinical resistance prevalence, we collected selected socioeconomic factors for all included countries from the World Bank database. Gross domestic product (GDP) and the proportion of the population living in urban areas correlated with each other and the factors related to basic living conditions; access to electricity, basic sanitation index and availability of clean drinking water were strongly correlated with each other (Suppl. Fig. 5). Owing to the correlations between the collected socioeconomical factors, only basic sanitation, GDP and proportion of urban population were used in modeling clinical resistance rates. Models based on these three socioeconomical factors performed better than models based on sewage data only (*R*^2^ 0.68–0.84, Suppl. Table 1). This showed that although being indirect measures of clinical resistance, socioeconomical factors correlated with clinical antibiotic resistance prevalence on country level, thus confirming the results by^16,17^.

**Fig. 4:**
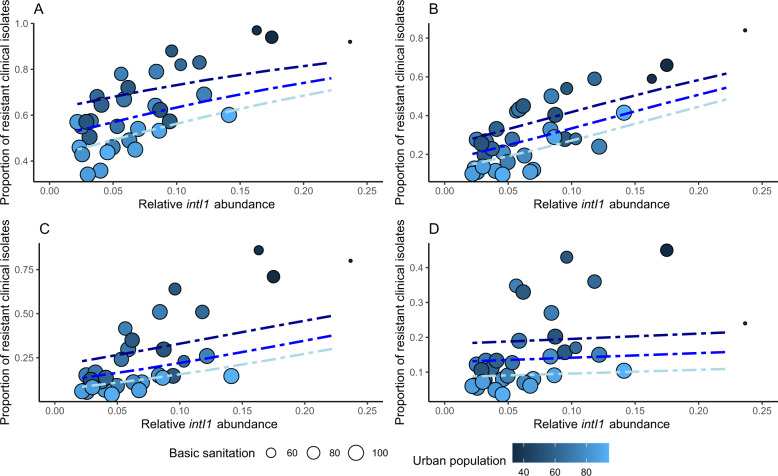
*E. coli* clinical resistance models based on combined sewage and socioeconomical data. Proportion of clinical resistant invasive *E. coli* isolates to aminopenicillins **a**, fluoroquinolones **b**, third-generation cephalosporins **c**, and aminoglycosides **d** against relative *intI1* integrase gene abundance in sewage. Proportion of urban population is shown with different shades of blue and size of point corresponds to basic sanitation index. The blue lines show the fitted clinical resistance from the beta regression model with socioeconomical factors and relative *intI1* abundance (see text for details). Three different socioeconomical scenarios (blue lines) have been fitted to the model to shown how sewage data correlates with clinical resistance. Dark blue line: lower quartile (25%), blue: mean quantile (50%) and light blue: upper quantile (75%) of socioeconomical factors fitted to the model. Note that GDP is not shown in the plots, but is included in all models and fitted lines.

**Fig. 5:**
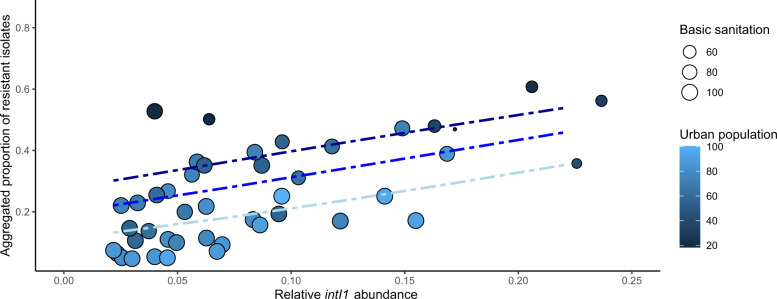
Aggregated resistance model based on combined sewage and socioeconomical data. Aggregated resistance index as described in Collignon et al.^16^ against relative *intI1* integrase gene abundance in sewage. Proportion of urban population is shown with different shades of blue and size of point corresponds to basic sanitation index. The blue lines show the fitted clinical resistance from the beta regression model with socioeconomical factors and relative *intI1* abundance (see text for details). Three different socioeconomical scenarios (blue lines) have been fitted to the model to shown how sewage data correlates with clinical resistance. Dark blue line: lower quartile (25%), blue: mean quantile (50%) and light blue: upper quantile (75%) of socioeconomical factors fitted to the model. Note that GDP is not shown in the plots, but is included in all models and fitted lines. Data from Nigeria and Peru are not included.

To explore if we could refine our models further, we proceeded by combining socioeconomic factors with our sewage-based models. The simplistic *intI1* model performed better or equally well as the different ARG models and was therefore used in the combined models. Relative *intI1* abundance in the sewage metagenomes, the logarithm of GDP, basic sanitation index and the proportion of urban population were included in all models of clinical resistance. The socioeconomical factors were negatively correlated with clinical resistance; more urban population, better sanitation and bigger GDP predicted lower clinical resistance rates, whereas higher sewage resistance predicted higher clinical resistance (Figs. 4 and 5). In all models, except for aminoglycoside resistance, a combined model with sewage data and socioeconomical factors gave a better model fit than using either alone (Table 1, Supplementary Table 1). Already in the sewage data alone model, the aminoglycoside resistance model had the lowest model fit and the combination with socioeconomic factors did not give a better model fit than using socioeconomic factors alone (Table 1 and Fig. 4d).

**Fig. 6:**
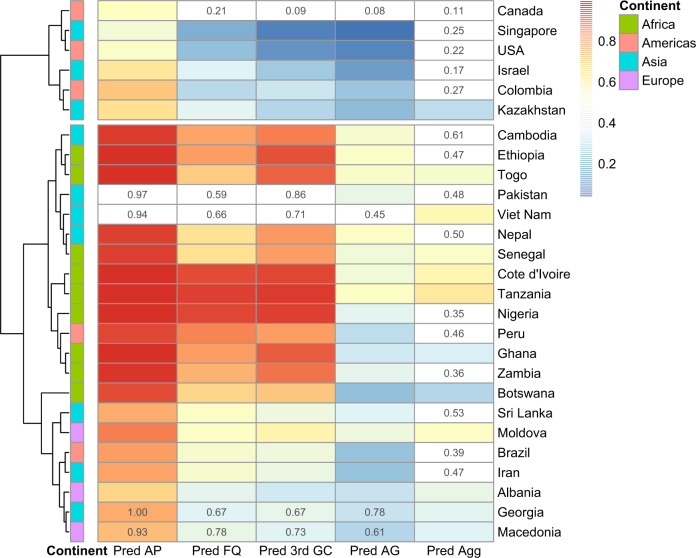
Predictions of clinical resistance prevalence using combined models. Predictions for countries where we did not have clinical resistance data from at least one of the resistance classes, including the aggregated resistance index, based on the final beta regression models including both relative abundance of relative *intI1* integrase gene abundance in sewage and socioeconomic data. In cases where there was clinical surveillance data for some antibiotic classes available, only the surveillance data are shown as proportion. The numbers shown for Georgia and Macedonia are based on very few (<100) isolates, so they were not included in the models, but were included in the predictions. The values from surveillance are displayed in top of the predicted ones. *Pred AP* predicted aminopenicillin, *Pred FQ* predicted fluoroquinolone, *Pred 3rd GC* predicted third-generation cephalosporin, *Pred AG* predicted aminoglycoside, *Pred Agg* predicted aggregated resistance index.

Based on the best combined models, we assessed how well clinical resistance could be predicted in countries where we did not have clinical surveillance data. To estimate how good the models are at predicting clinical resistance, a leave-one-out cross-validation was performed. From the cross-validation, the mean absolute error (MAE) was calculated for each resistance profile (aminopenicillin, fluoroquinolone, third-generation cephalosporins, aminoglycoside and aggregated resistance index). In general, all models performed well in predicting clinical resistance with the MAE ([%]) ranging from 5.99 (aminopenicillin) to 8.27 (fluoroquinolone) (Methods, Supplementary Table 2, Supplementary Fig. 6). Next, using the combined models with socioeconomic factors and sewage data for each resistance class, we predicted the proportions of resistant clinical isolates for all countries where clinical resistance data were lacking from any of the classes included in this study. Not surprisingly, the models predict the highest resistance rates for countries in Africa, South Africa being an exception (Fig. 6). For Georgia and Macedonia, the clinical resistance data was based on very few isolates (<100), so these countries were not included in the original models. However, our models predict lower clinical resistance levels in these two countries than the surveillance data, suggesting that the data based on only a few isolates was potentially biased towards more severe clinical cases and does not reflect the real prevalence of clinical resistance in these countries (Fig. 6). As stated earlier, Nigeria and Peru seemed to be outliers with high *intI1* abundance in sewage, but only moderate aggregated resistance index. Our combined models predict very high clinical resistance levels in Nigeria and Peru (Fig. 6). There is also support in the literature for the higher clinical resistance levels for these two countries^20,21^. Clinical resistance data obtained from ResistanceMap^22^ for Nigeria shows a high level resistance in *E. coli* against third-generation cephalosporins and fluoroquinolones (>75% in 2017), in line with the predictions made by our model. Resistance data for Peru is lacking from ResistanceMap.

**Fig. 7:**
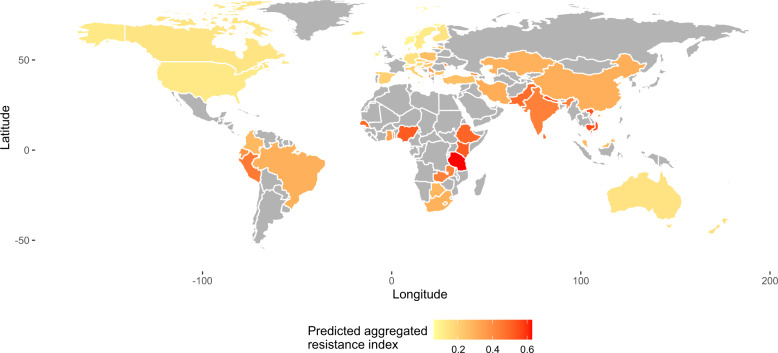
Global predictions for aggregated resistance index. Yellow means lower resistance and red higher resistance levels. Countries in gray have no predictions owing to missing sewage metagenomic and/or socioeconomic data.

To get an overview of the global clinical resistance we used the model for the aggregated resistance index (from ref. ^16^) for all countries where sewage resistance and socioeconomical data were available (Fig. 7, for other classes see Supplementary Fig. 7). Our global predictions were in line with previous literature and showed that the clinical resistance levels are highest in South America, Africa, and Asia^16,23^. Of course, these are predictions rather than direct observations. Although the leave-one-out cross-validation added confidence to the predictive power of the model, and could reflect accurately the overall clinical resistance situation in these countries, values should be interpreted with caution.

Sewage metagenomic data certainly have limitations and cannot directly replace clinical surveillance. For example, current methods are unable to link resistance genes found in sewage metagenomes to their bacterial hosts. As resistance patterns in different pathogens are correlated (Supplementary Figs. 9–12) it is plausible that the resistance of additional species also could be predicted by sewage metagenomics data, but such models would need further optimization and validation. With our models, some resistance patterns are better predicted than others and the results most likely will provide more information about the overall prevalence of antibiotic resistance than giving a high resolution of specific resistance profiles for a given species. As mentioned, many resistance phenotypes are a result of point mutations, and detecting these with a metagenomics approach is challenging. From a conceptual point of view, isolate-based approaches are therefore likely needed for sufficient precision for all classes of antibiotics. A metagenomic approach could still provide important and cost-efficient information on geographical and temporal trends, thereby indicating where stewardship actions are effective, ineffective or particularly needed.

In conclusion, sewage data alone correlated to the overall clinical resistance levels. However, despite efforts to use the information from the abundances of separate ARG classes, the correlations to clinical resistance did not improve. A model based only on relative abundance of *intI1* performed better or on par with all tested approaches based on ARGs. Models based on sewage metagenomic data had, furthermore, a lower model fit than models based on socioeconomic factors. One important reason for this is likely the differences in bacterial populations between those causing disease and those contributing to gene counts in the sewage metagenomes, e.g., not all *E. coli* strains cause disease in humans. Another reason is the difficulties to translate abundances of disconnected single resistance genes into bacterial phenotypes, including the inability to detect mutation-based resistance. There are also differences in the human populations covered, as sewage is usually from one or a few cities covering all inhabitants including carriers without disease, whereas clinical resistance data are usually generated on a country-wide basis but with a bias towards hospitalized, sick patients. In here, the sewage data were from one to few sewage treatment plants and then extrapolated to represent the whole country, as the clinical data were on country level. Optimal data would include time-matched, clinical resistance data representing the same population served by the sewage treatment plant. The best models were achieved by combining sewage metagenomic data with socioeconomic data. Such a model could, as demonstrated here, be used for clinical resistance prevalence predictions to get a view of global clinical resistance prevalence including countries where surveillance data on clinical resistance is lacking.

## Methods

### Data extraction

All data used in this study were retrieved from public databases or published articles (Table 2).

**Table 2:** Sources and descriptions of data used in this study.

Data source	Data description and links to data
Hendriksen et al.^15^	The raw sequence data from this study was downloaded from ENA under project ERP015409. The data consists of 234 sewage metagenomemes from 62 countries. The antibiotic resistance genes were annotated against ResFinder v.3.1.0 database and *intI1* against MobileGeneticElementDatabase. The third-generation cephalosporin ARGs were determined using a list of third generation cephalosporin drugs obtained from Medscape. The names of the drugs were then matched with the resistance profiles provided by ResFinder.
	ResFinder: https://bitbucket.org/genomicepidemiology/resfinder
	Medscape: https://reference.medscape.com/drugs/cephalosporins-3rd-generation
	MobileGeneticElementDatabase: https://github.com/KatariinaParnanen/MobileGeneticElementDatabase
Collignon et al.^16^	Aggregated resistance index. This index includes data on *Escherichia coli* and *Klebsiella spp* resistance to third-generation cephalosporins, fluoroquinolones, and carbapenems, and methicillin-resistant *Staphylococcus aureus*.
EARS-Net, CAESAR, ResistanceMap	Clinical resistance data for invasive *E. coli* (blood, CSF) against four different classes of antibiotics, i.e., aminopenicillins, fluoroquinolones, third generation cephalosporins and aminoglycosides, were extracted. Only data from >100 isolates was considered reliable and included in the actual analyses. Clinical resistance data are available from 36 countries. For some countries clinical resistance data was not available for all antibiotic classes (Supplementary Table 3).
	**EARS-Net**: https://www.ecdc.europa.eu/en/antimicrobial-resistance/surveillance-and-disease-data/data-ecdc
	**CAESAR**: https://www.euro.who.int/en/health-topics/disease-prevention/antimicrobial-resistance/publications/2018/central-asian-and-eastern-european-surveillance-of-antimicrobial-resistance-annual-report-2018-2018
	**ResistanceMap**: resistancemap.cddep.org
The World Bank	Data on socioeconomic factors were extracted from The World Bank Databank (https://databank.worldbank.org/source/health-nutrition-and-population-statistics) i.e., GDP, basic sanitation, basic drinking water, urban population, access to electricity

### Metagenome sequence analysis

Raw sequencing data were downloaded from ENA under the project accession ERP015409. Remaining adapter sequences were removed with cutadapt v. 2.7^24^. The trimmed reads were searched for ARGs against the ResFinder database v.3.1.0^25^ using DIAMOND v. 0.9.l14^26^ with parameters “–*id 90 –min-orf 20 –seq no*” and the ARG counts in each sample were normalized with the total sequencing effort (M bases). Sum of normalized gene counts was used as a proxy for sewage resistance load. The *intI1* integrase gene abundance was determined in similar fashion (except “—id 95”) using the MobileGeneticElementDatabase from ref. ^27^ and adding up the *intI1* integrase gene counts. For countries with more than one metagenome, the mean of counts was used.

To determine which ARGs that were connected to *E. coli*, all genomes annotated as *E. coli* were downloaded from NCBI genbank in December 2019. The genomes were then searched for ARGs against the Resfinder database using DIAMOND v. 0.9.14 with parameters “*blastx –max-target-seq 0 –subject-cover 70*”. The best scoring hits with a sequence identity higher than 98% at a given position in the genome were then extracted and used in the subsequent analysis. The most common ARGs in the available *E. coli* genomes were determined through counting the occurrence of each extracted hit.

### Statistics and reproducibility

The prevalence of clinical resistance was modeled using beta regression with log-log link function using function *betareg* from **betareg** v. 3.1–2. We used the pseudo *R*^2^ values calculated by *betareg* function and referred to them as *R*^2^ values for clarity throughout the manuscript. The difference in model fit between nested beta regression models were assessed using likelihood ratio test with function *lrtest* from package **lmtest** (v.0.9.37). The PCA ordinations were calculated using function *rda* from **vegan** v. 2.5–6. The correlation plots were drawn using function *ggpairs* from **GGally** v.1.4.0. The heatmap was produced with package **pheatmap** v. 1.0.12. All other figures were produced with **ggplot2** v. 3.2.1.

The leave-one-out cross-validation was performed as follows. For each type of clinical resistance, the sewage data together with the socioeconomic data were modeled as above but with one country excluded. The level of clinical resistance was then predicted for the excluded country using the obtained model. This was repeated for each country in the data set. For each country the absolute error, consisting of the absolute value of the difference of the real value and the predicted value, was calculated. The mean absolute error (MAE) was then calculated based on the average over the absolute error for all countries.

All data analysis steps were done in **R** v. 3.6.2. For more-detailed data analysis see the project analysis repository at: www.github.com/karkman/GlobalSewage. The project repository also contains all needed numerical data to reproduce the analyses in this project.

### Reporting summary

Further information on research design is available in the Nature Research Reporting Summary linked to this article.

## Supplementary information

Supplementary Information

Reporting Summary

## Data Availability

The data sets analyzed during the current study are publicly available and listed under Data extraction in Methods.

## Code availability

Detailed data analysis for this study can be found from: www.github.com/karkman/GlobalSewage.
