# Supplementary material for: Predicting clinical resistance prevalence using sewage metagenomic data
Source: Commun Biol. 2020 Nov 26;3:711. doi: 10.1038/s42003-020-01439-6 (PMC7692497; doi:10.1038/s42003-020-01439-6)
Supplement: Supplementary file 1 — Supplementary Information [file 42003_2020_1439_MOESM1_ESM.docx]

**Supplementary**

**Supplementary Table 1.** Model fit for socioeconomical factor only models

|  | R^2 | MAE |
| --- | --- | --- |
| AP-res | **0.84** | **7.07** |
| FQ-res | **0.72** | **8.80** |
| 3GC-res | **0.84** | **7.35** |
| AG-res | **0.68** | **5.98** |
| Avg-res | **0.68** | **7.85** |

**Supplementary Table 2.** Results from leave-one-out cross-validation for respective final model for each resistance profile. The models were based on the *intI1* count, the proportion of urban population, the logarithm of GDP and the level of basic sanitation.

|  | Aminopenicillin | Fluoroquinolone | 3^rd^ gen. cephalosporins | Aminoglycoside | Aggregated resistance |
| --- | --- | --- | --- | --- | --- |
| σ_clinical resistance_ [%] | 16.80 | 18.29 | 22.58 | 11.29 | 16.24 |
|  |  |  |  |  |  |
| *Model performance* |  |  |  |  |  |
| Pseudo R^2^ | 0.85 | 0.80 | 0.86 | 0.68 | 0.76 |
| Mean Absolute Error [%] | 5.99 | 8.27 | 6.79 | 6.23 | 7.04 |

**Supplementary Table 3.** Data sources for *E. coli* clinical resistance data

| Country | *E. coli* data source (2016) | Note |
| --- | --- | --- |
| Australia | ResistanceMap |  |
| Austria | EARS-Net |  |
| Bulgaria | EARS-Net |  |
| Canada | ResistanceMap | 2014 data |
| Switzerland | CEASAR |  |
| China | ResistanceMap |  |
| Czech Republic | EARS-Net |  |
| Germany | EARS-Net |  |
| Denmark | EARS-Net |  |
| Ecuador | ResistanceMap |  |
| Spain | EARS-Net |  |
| Finland | EARS-Net |  |
| Georgia | CEASAR |  |
| Croatia | EARS-Net |  |
| Hungary | EARS-Net |  |
| India | ResistanceMap |  |
| Ireland | EARS-Net |  |
| Iceland | EARS-Net |  |
| Italy | EARS-Net |  |
| Luxembourg | EARS-Net |  |
| Latvia | EARS-Net |  |
| Macedonia | CEASAR |  |
| Malta | EARS-Net |  |
| Malaysia | ResistanceMap | 2015 data |
| Netherlands | EARS-Net |  |
| Norway | EARS-Net |  |
| New Zealand | ResistanceMap | 2015 data |
| Poland | EARS-Net |  |
| Pakistan | ResistanceMap | 2017 data |
| Serbia | CEASAR |  |
| Slovakia | EARS-Net |  |
| Slovenia | EARS-Net |  |
| Sweden | EARS-Net |  |
| Turkey | CEASAR |  |
| Viet Nam | ResistanceMap |  |
| South Africa | ResistanceMap |  |

**Supplementary Figure 1.** ﻿Correlation between different clinical resistance patterns

**
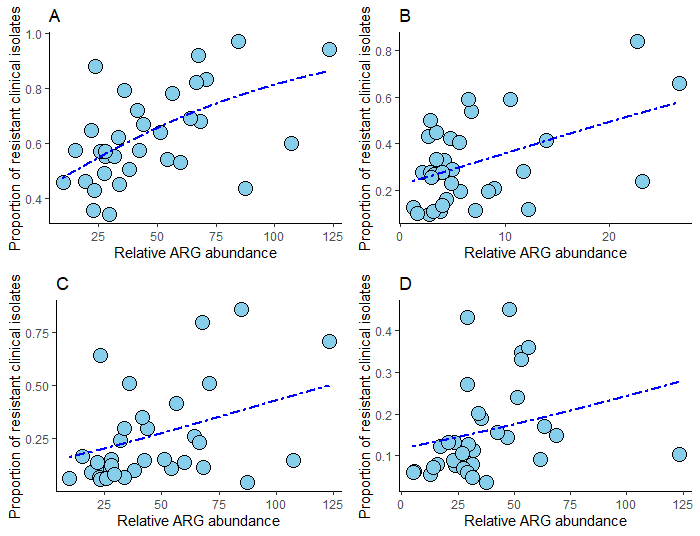
**

**Supplementary Figure 2.** Proportion of resistant invasive E. coli clinical isolates to aminopenicillins (A), fluoroquinolones (B), 3rd generation cephalosporins (C) and aminoglycosides (D) against the relative abundance of ARGs of the respective gene class; (A): beta-lactamases, (B): fluoroquinolone resistance genes, (C): beta-lactamases and (D): aminoglycoside resistance genes). The blue line shows the fitted clinical resistance from the beta regression model with respective resistance gene class abundance as explanatory variable.


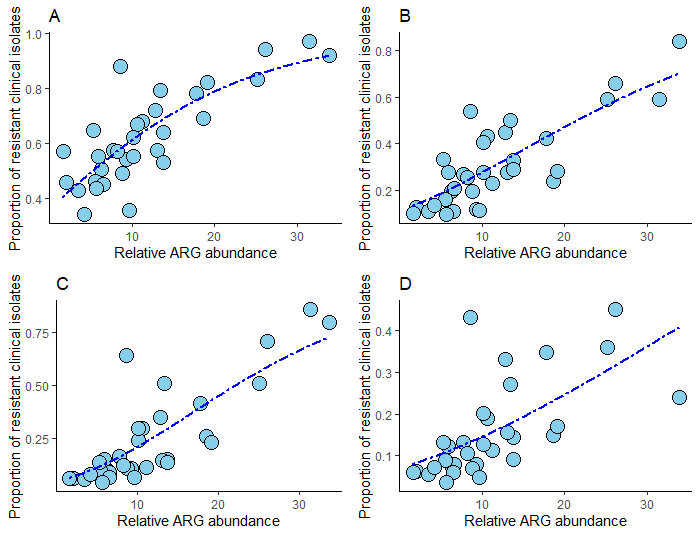


**Supplementary Figure 3.** Proportion of resistant invasive E. coli clinical isolates to aminopenicillins (A), fluoroquinolones (B), 3rd generation cephalosporins (C) and aminoglycosides (D), with the outlier Malta removed from the analysis, against the relative abundance of the 10 most prevalent ARGs in E. coli. The blue line shows the fitted clinical resistance from the beta regression model with the relative ARG abundance as explanatory variable.


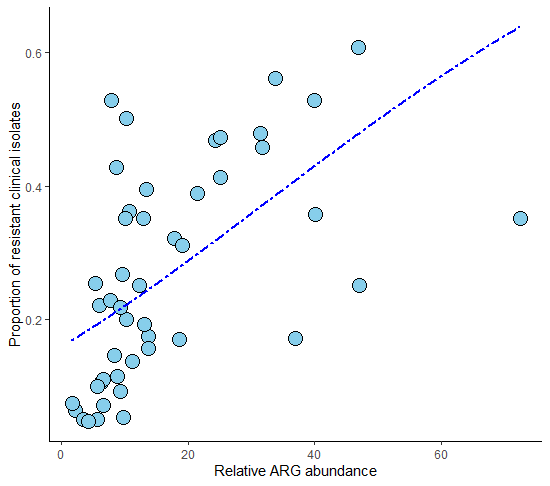


**Supplementary Figure 4.** Aggregated resistance index against the relative abundance of the 10 most prevalent ARGs in *E. coli*.

**Supplementary Figure 5.** Correlations between socioeconomical factors


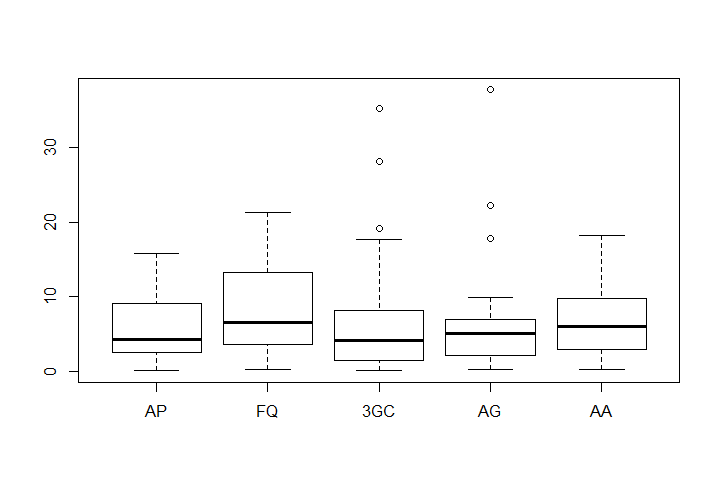


**Supplementary Figure 6.** Boxplot of the absolute errors from the leave-one-out cross-validation of the final models including the sewage data.

**Supplementary Figure 7.** Global predictions for clinical aminopenicillin (A), fluoroquinolone (B), 3^rd^ generation cephalosporin (C) and aminoglycoside (D) resistance.

**Supplementary Figure 8.** Correlations between socioeconomical factors, clinical resistance and sewage markers.

**Supplementary Figure 9.** Clinical aminopenicillin resistance prevalence in *E. faecalis, E. faecium* and *E. coli.* Upper triangle shows Pearson correlations between different pathogens (2016 data from EARS-Net).

**Supplementary Figure 10.** Clinical fluoroquinolone resistance prevalence in *Acinetobacter spp, E. coli, K. pneumoniae* and *P. aeruginosa.* Upper triangle shows Pearson correlations between different pathogens (2016 data from EARS-Net).

** Supplementary Figure 11.** Clinical 3^rd^ generation cephalosporin resistance prevalence in *E. coli* and *K. pneumoniae.* Upper triangle shows Pearson correlations between different pathogens (2016 data from EARS-Net).

**Supplementary Figure 12.** Clinical aminoglycoside resistance prevalence in Acinetobacter spp., E. coli, K. pneumoniae and P. aeruginosa. Upper triangle shows Pearson correlations between different pathogens (2016 data from EARS-Net).
